# Low-Frequency Sound Pressure and Transtympanic Endoscopy of the Middle Ear in Assessment of “Spontaneous” Perilymphatic Fistula

**DOI:** 10.5402/2012/137623

**Published:** 2012-08-07

**Authors:** Ilmari Pyykkö, Ziane Selmani, Jing Zou

**Affiliations:** Department of Otolaryngology, University Hospital of Tampere, University of Tampere, Teiskontie 35, 33521 Tampere, Finland

## Abstract

This study was designed to verify an eventual perilymphatic fistula (PLF) in 264 patients with sensorineural hearing loss (SNHL) and/or vertigo. The patients were exposed to a low-frequency sound stimulation (LFS) on posturography to objectively test Tullio's phenomenon and Hennebert's sign. Endoscopes with 5 degree and 25 degree of visual angle and an outer diameter of 1.7 mm were used. The round window niche, with its foldings, oval window with stapes superstructure, a part of the facial recess and the area in the fissula ante fenestram were examined and video recorded. In one patient, we endoscopically verified a fistula in the round window membrane (resulting from a diving accident) that was covered with a fibrinous layer. In 4 cases, there was abnormal mucosal shining in the round window but without PLF. In 7 cases, the tympanic cavity could not be visualized because of the adhesive middle ear process, the abnormal anatomy, or the prominent exostoses of the ear canal prohibited vision. In 34 patients, LFS provoked unsteadiness on posturography without PLF. In 6 cases, a postoperative middle ear infection was recorded. No permanent tympanic membrane perforation occurred. It is unlikely that disease entity of “spontaneous PLF” exists. Tympanoscopy should be regarded as the first choice when a PLF is suspected.

## 1. Introduction

Perilymphatic fistula (PLF) is an abnormal communication between the middle ear and the perilymphatic space, mostly due to laceration of the oval and/or round window, or congenital defects in the area of fissula ante fenestram or fossula post fenestram [1]. PLF may be caused by external injury such as head trauma, barotrauma, physical exertion such as straining, nose blowing, sneezing, among others [2]. Some diseases such as Mondini malformation, large cochlear aqueduct syndrome [3], and middle ear abnormalities are linked to PLF [4], among others. When no obvious reasons for PLF exists, it is called spontaneous [5]. This does not mean that spontaneous PLF would occur without trauma, but the trauma may be so minute that it is not recognised. Idiopathic PLF has been implicated as one of the etiological factors associated with idiopathic sudden or fluctuating sensorineural hearing loss and vertigo, which occurs in defined diseases such as Meniere's disease, otosclerosis, and sudden deafness, among others [6].

So far, there are no objective tests that can exclude or verify a PLF. Thus, the diagnosis of PLF is very symptom-oriented. Clinically, PLF is suspected when the patient complains of the Tullio's phenomenon [7, 8]. Most commonly the Hennebert's sign is evaluated, in which test the posture and eye movements are inspected when the ear canal pressure is slowly increased or decreased [6]. The test has been modified and performed with impedance tympanometry in ENG, [9] or on posturography [10–12]. The validity of these tests in assessing a PLF is not without controversy [8].

Early diagnosis may preserve the hearing and prevent vestibular function loss of patients with PLF [13]. Detection of PLF has so far been restricted to a high degree of suspicion. Explorative tympanotomy is often carried out to visualize an eventual PLF. Nevertheless, direct inspection of the middle ear by elevating the tympanomeatal flap may lead to erroneous conclusions when PLF is suspected [14]. Poe et al. [15] demonstrated that middle ear processes can be imaged endoscopically by introducing a small-diameter rigid endoscope through the tympanic membrane. The windows and mucosal membranes can be inspected to exclude perilymphatic leaks [14, 16].

The purpose of the present study was to evaluate various vestibular and cochlear disorders in order to determine the presence of PLF. The endoscopy was carried out through a myringotomy incision made under local anesthesia. All examinations were performed in an office setting. We also wanted to study the relevance of sound and pressure loading on posturography in assessing of PLF.

## 2. Subjects and Methods

### 2.1. Subjects

We investigated altogether 264 subjects out of patients with various inner ear diseases clinically susceptible to PLF. For this study, we studied the middle ear by transtympanic endoscopy and by evaluation of Tullio's phenomenon. The age of the subjects varied from 22 to 80 years (mean age 48 years). The final diagnoses of the subjects are shown in Table 1.

### 2.2. Methods

Audiometry was done in all cases before examination, and one month after tympanoscopy. In connection to tympanoscopy, we evaluated the electrocochleography by using round window electrodes and also cochlear blood flow by using a laser Doppler flux meter placed on the promontorium. The results have been published elsewhere [17].

The investigation started with evaluation of the vestibule-spinal responses when loading the ear with low-frequency sound (LFS). Both ears were studied separately. The low-frequency sound (LFS) test was used to evoke abnormal body sway which was measured on posturography [8]. The LFS was generated with a piston type air sampling pump (MSA, USA), which has an adjustable stroke frequency ranging from 6 to 80 Hz. The frequencies used in the test were 25, 50, and 63 Hz at a sound pressure level of 130, 132, and 135 dB, respectively.

The patient was then placed under the operation microscope in the Trendelenburg position. A part of the tympanic membrane (about a 1 mm wide area extending from the annulus to the umbo) was anaesthetized with 90% phenol solution. After a radial paracentesis of about 5 mm in length we had a bloodless view into the posterior tympanic cavity. An endoscope (1.7 mm in diameter, 120 mm in length) with an angle of 5 deg (Wolff GmBH, Germany) was inserted into the tympanic cavity. A general view into the tympanic cavity was made. Thereafter, the round window was examined. A forceful Valsalva maneuver and the Queckerstedt test were performed. Any fluid collection was recorded. The 25 degree tympanoscope was introduced and the oval window area was examined paying special attention to the fissula ante fenestram. The Valsalva maneuver and the Queckerstedt test were repeated. A high suspicion for PLF was kept in mind. The procedure was video-recorded and replayed if necessary.

## 3. Results

### 3.1. Tympanoscopy

The tympanic cavity could be visualized in all except seven subjects. In one subject who had undergone stapes surgery, scar tissue formation was so prominent that the oval window and prosthesis area could not be identified. In one subject, the anatomy of the middle ear was changed so that the oval window was situated more inferior than usual and the bone rim of the round window niche was flattened, obstructing vision through the window. In two patients, the tympanic cavity could not be visualized because of retraction of the tympanic membrane. In three subjects, the exostoses and bleeding from the bony canal hindered the inspection. In most subjects, there was folding in the round window that occluded direct viewing of the window membrane. In about 35% of the cases, we could identify the whole round window membrane. All patients were discharged home immediately after the procedure.

Usually the best way to visualize the round window was to examine it with a 5 deg endoscope. Although the angle of the endoscope was not optimal, the best area visualized by this scope was the round window area. Sometimes the round window could be examined with a 25 degree scope, but usually the round window niche and hypotympanic space were too narrow to allow insertion of the 25 degree scope into the area.

The oval window area was best inspected with the 25 degree scope. Excellent visibility was achieved in the fissula ante fenestra area. The stapes footplate was inspected for any abnormalities or leaks. The visibility in the posterior part of the stapes footplate was limited, and especially the posterior crus-facial nerve rim limited visual inspection.

Inspection of the patient in a recumbent position with head down (trendelenburg position) was used to provoke an increase in the perilymph flow through an eventual PLF. We also performed a forceful Valsalva maneuver and the Queckerstedt test. These examinations were carried out twice, once for examination of the round window and once for that of the oval window.

We were able to verify one traumatic fistula in the round window. It was covered with fibrinous membrane and caused by a diving accident. In one case, there was abnormal mucosal shining in the round window niche and oval window area that could signify a PLF. Three cases with abnormal shining around the round window probably exhibited reactive changes to tympanoscopy. In two cases, we applied tissue glue to the area, but the symptom was not relieved. One case was followed up without operation and showed uneventful course.

### 3.2. LFS Stimulation Test

**Figure 1:**
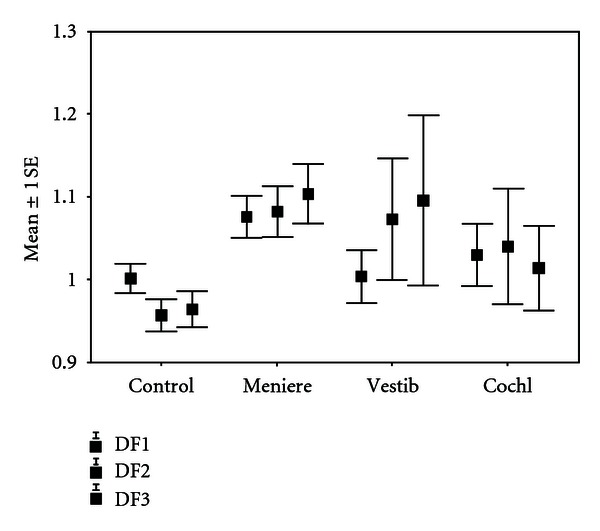
Effect of low frequency sound on postural stability in normal controls, patients with Meniere's disease, patients with vestibulopathy of unknown etiology, and in patients with cochleopathy of unknown etiology. Means and standard error of the means are given for the stimulation frequencies of 25, 50 and 63 Hz at a sound pressure level of 130, 132 and 135 dB, respectively.

Figure 1 shows the outcome of the fistula test at different stimulation frequencies in four categories of the patients. In the analysis of variance, the groups differed significantly in their responses to LFS stimulation. Patients with Meniere's disease responded by increased body sway to LFS stimulation. Thus, 24 of the 149 patients with Meniere's disease had abnormal LFS responses. The corresponding numbers for vestibulopathy were 5 out of 53, cochleopathy 3 out of 32, sudden deafness 2 out of 14. In those 34 patients in whom the LFS stimulation provoked unsteadiness in posturography analogous to the Tullio's phenomenon, no presence of PLF in tympanoscopy could be verified.

### 3.3. Complications Linked to Procedure

No difference was observed in the hearing threshold before and after the tympanoscopy. All endoscopies were well tolerated by the patients, except in 3 cases, in whom the exostoses of the bony canal caused difficulties with bleeding and discomfort so that the examination has to be interrupted. These patients found the procedure painful when the endoscope was in contact with the skin of the ear canal. Six postoperative infections occurred and were treated successfully. All myringotomies were fully healed at the time of followup. In one patient, healing of the perforation took more than two months before it was closed. Five patients experienced a reduction in their ability to taste, but the symptom subsided 2 to 4 weeks after endoscopy.

## 4. Discussion

Endoscopy has had a rather limited success in otology when compared with the advances in endoscopy techniques in rhinology or other disciplines of surgery. Otoendoscopy was introduced by Nomura [18] and Takahashi et al. [19] for documentation purposes. In otosurgery, it has been proposed for second look mastoidectomy, [20] in middle ear surgery, in the verification of middle ear recesses in conjunction with cholesteatoma surgery, [21, 22] in hearing preservation surgery of vestibular schwannomas [23], and in inspection of the tympanic cavity [22]. The use of endoscopy in the detection of PLF has been promoted by Poe et al. [15] They demonstrated that, after the use of local anesthetics for elevation of a tympanomeatal flap, oozing of exudate occurred in the middle ear, leading to a false diagnosis of PLF in a patient who had normal structures in tympanoscopy [15, 24]. Furthermore, they demonstrated that in three cases with experimental PLF, the findings could be verified in middle ear microscopy as well as in tympanoscopy [15, 16]. Provocation maneuvers, such as Queckerstedt's manoeuvre and the forced Valsalva test are easier to perform in tympanoscopy than in otomicrosopy. Nevertheless, it has been suggested that the accuracy of endoscopy is not sufficient for detecting minute “hidden or submucous PLFs.” This holds for all present examination methods, where nonopen fistulas cannot be verified. We demonstrated in guinea pig with artificial fistula that the optic resolution was satisfactory whereas on the video screen resolution was not satisfactory (unpublished observation). Ogawa et al. [25] demonstrated that, in one patient finally diagnosed with idiopathic PLF, tympanoscopy could not reveal the fistula. In spite of this event, these authors recommend the use of tympanoscopy for diagnosing idiopathic PLF. Perhaps the approach suggested by Kohut et al. [6] is more accurate: the middle ear mucosa is elevated and coagulated with a KTP laser. So far, without any relevant objective test for PLF, it is difficult to know which patient should be operated on. Hitherto tympanoscopy seemed to be the golden rule for the detection of PLF on which all diagnostic tests or operative findings should be benchmark. Tympanoscopy is an ambulatory procedure with very few complications and it seems to have high diagnostic accuracy for PLF [16].

### 4.1. Diagnosis of PLF

One of the most disturbing things in assessing diagnosis of PLF is that there are no characteristic signs, symptoms, or a golden rule for verifying of PLF. There are no reliable diagnostic tests available [26]. As indicated by Kohut et al. [6], the symptoms of PLF coincide with those present in a majority of cases with Meniere's disease. The observation of endolympahic hydrops in cases with PLF may in fact be the reason for Meniere-like symptoms that may be secondary to PLF. Several objective methods for the detection of PLF have been advocated. Fluorescein applied intrathecally may assist to reveal a fistula better than fluorescein applied intravenously [27] as this has not proved to be reliable for the detection of PLF [28]. During surgery, for the detection of PLF mineral, oil droplets have recently been advocated to trap the perilymph and to visualize the leak [29]. Also, beta2-transferrin has been advocated [30, 31] A two-dimensional polyacrylamide gel electrophoresis has been studied in the verification of a PLF [32]. The value of these tests remains to be documented. Intrathecal fluorescein can be visualized with an otoendoscope equipped with proper filters. It is possible that in these instances flexible endoscopes can be utilized [15, 33].

The history and symptoms of PLF can be variable [26]. There is general consensus that a trauma to the ear, either explosive or implosive, is the most important factor in the etiology of PLF. It has been debated whether a disease called “idiopathic or spontaneous PLF” actually exists [5]. Shea [34] criticised the occurrence of idiopathic PLF and could not verify an idiopathic PLF in his large series of otologic patients. In a randomised, retrospective study, Kohut et al. [6] examined with microscopy a series of temporal bones. The authors defined objective criteria based on symptoms of PLF. These were “Sudden or fluctuate hearing loss or slowly progressive hearing loss and the vestibular symptoms were equivalent to positional vertigo or constant disequilibrium.” A cornerstone in the diagnosis was a positive fistula test (Hennebert's sign or symptom or equivalent). With blind assessment of the temporal bones, they could verify with high specificity (91%) and moderate sensitivity (59%) a PLF. It was noteworthy that a majority of the patients had a symptom entity equivalent to Meniere's disease. Their clinical approach was much the same as ours, in which we examined with tympanoscopy all those patients with unconfirmed diagnosis having a high suspicion of PLF during a four-year course. We were able to confirm the presence of one PLF in the round window after a diving accident. In three additional cases, abnormal mucosal shining with slight oozing was observed in the fossula post fenestra area. These cases were considered possible candidates for a PLF, but closer examination and absence of fluid oozing during provocation prompted rejection of the diagnosis of PLF. The slow accumulation of fluid was probably due to mechanical irritation of the middle ear mucosa [24].

Kohut et al. [35] demonstrated the histopathologic findings of a temporal bone in idiopathic PLF, as fissure tracts in fissula ante fenestram which contained loose connective tissue, and which are composed of fibroblasts that are not densely packed, and between them are large intercellular spaces. They also reported that the submucosa of the fissure of the round window niche was composed of dense collagen next to bone, and extended over the orifice, with components extending into the lumen of the fissure. It remains to be documented whether these tiny openings are able to cause the various, prominent symptoms in the patients, and also by which mechanism the symptoms arise. These fissure tracts cannot be naturally visualized with an endoscope.

### 4.2. Complications Linked to Tympanoscopy

Risks associated with the tympanoscopy procedure are minimal, and can be as slight as inconvenience caused by dizziness due to thermal heating of the lateral semicircular canal [36] or may result in postoperative infection, lesion to chorda tympani, or permanent ear drum perforation. So far, no ossicular chain luxation has been reported. In this series, the postoperative infection rate was about 2% but with a careful technique it can be reduced, by avoiding the endoscope from contacting the ear canal wall. Subjects with a history of chronic middle ear infection with thin secondary tympanic membrane were the most susceptible to infection in the present study. Perhaps patients with a large secondary membrane should not be examined by endoscopy through the tympanic membrane.

Five patients had a temporary reduction in taste that was probably linked to the phenol used for the anesthesia of the ear drum. Other alternative topical anesthetics are tetracaine base powder dissolved in isopropyl alcohol [37] or racemised lidocain-prilocain emulsion (EMLA, Astra, Sweden) applied for 15 minutes to the ear drum, or use of iontophoresis with 4% lidocaine solution with 1 : 1000 epinephrine [15]. These anesthetics may lack effect on the chorda, but do not provide as bloodless entry into the tympanic cavity as does phenol. Furthermore, by using phenol, only the paracentesis streak must be anesthetized, not the whole ear drums, as with the other drugs. We are aware that permanent perforations do occur after tympanoscopy, but the probability seems to be rather low, since in our 264 cases no permanent perforation has occurred.

### 4.3. Low-Frequency Sound Stimulation and the Inner Ear

The vestibulespinal responses during LFS stimulation in the present study are similar to the vestibular responses found in Tullio's phenomenon. In Tullio's phenomenon, the sound pressure induces electrical potentials equivalent to cochlear microphonics from different receptors of the vestibular labyrinth [38]. Nine subjects had a history of Tullio's phenomenon. We could not relate the Tullio's phenomenon to any pathology [8] except endolymphatic hydrops, that could provoke Tullio's phenomenon with a similar mechanism as the Hennebert's sign, that is, anatomical connections between utriculus and the stapedial footplate [39]. Nevertheless, Minor et al. [40] observed Tullio's phenomenon in a patient with a dehiscence of the superior semicircular canal, first observed in computerized tomography and later verified in surgery. In the present stimulation mode, the pump also generated an underpressure in the ear canal that was pulling the ear drum and stapes laterally. In this respect, the LFS stimulation also generates test conditions that provoke the Hennebert's sign [8]. Hennebert observed that an underpressure and overpressure of the ear canal is able to cause nystagmus and instability in patients who have luetic softening of the bony capsule [41]. Both Tullio's phenomenon and the Hennebert sign were originally thought to be an indication of a fistula in the bony labyrinth. Later, it was concluded that also patients with PLF often show a positive Hennebert's sign, and the test has at present been commonly advocated to detect PLF [6, 8]. By using fluctuate air pressure loading in the outer ear canal, Wall and Casselbrant [12] demonstrated in animal model that, in PLF, pressure caused eye deviations and nystagmus. The responses were stronger at higher frequencies than at lower ones.

The number of positive test results in the LFS loading test on posturography in patients with Meniere's disease or vestibulopathy was unacceptably high, although the magnitude of the positive responses was significantly lower than in PLF patients [8]. Shepard et al. [11] performed by posturography a pressure loading test, and compared the outcome of the test in surgery conducted by two teams. The sensitivity between the teams varied from 53 to 100% and specificity from 56 to 89% for the verification of PLF. Black et al. [10] used posturography and reported a high hit rate of positive fistula tests (97%) in the vestibulospinal responses of 64 patients. Recently, Fitzgerald [42] has confirmed the value of the platform fistula test. Even with the present PLF method, the lack of specificity may not totally disqualify the value of the test. The accuracy of the fistula test varies even more in most of the tests used to detect a PLF. Supance and Bluestone [43] reported a positive fistula test in 54% of their surgically confirmed fistula cases. Singleton [44] reported that only 26% of their fistula patients had positive test results. The false-positive rate was 18%. Healy et al. [45] reported positive fistula test rate in 37% of their surgically confirmed cases. Seltzer and McCabe [2] reported a positive fistula test rate of 36% among 91 surgically confirmed fistulas. Finally, we found that Tullio's phenomenon test was not specific for PLF, thus tympanocopy is recommended as the first choice to perform when the PLF is suspected, especially during the acute phase of the disease before the fibirinous reaction can occur. Once the diagnosis of PLF is established, then tympanotomy could be then decided.

## Figures and Tables

**Table 1:** Endoscopic diagnosis of the subjects.

Diagnosis	Number (*n* = 264)
Meniere's disease	149
Vestibulopathy (vertigo)	53
Cochleopathy	
Progressive hearing loss with/or without tinnitus	32
Sudden deafness	13
Stapes surgery	16
PLF	1
